# A case report of liver infiltration from a large renal cell carcinoma: Diagnostic and management enigma

**DOI:** 10.1016/j.ijscr.2023.109045

**Published:** 2023-11-14

**Authors:** Devendra Choudhary, Maktum Naik, B.G. Vageesh, Anil Agarwal

**Affiliations:** Department of Gastrointestinal Surgery, Govind Ballabh Pant Institute of Medical Education and Research, New Delhi, India

**Keywords:** RCC, Right hepatectomy, Nephrectomy, Oligometastatic, Margin negative resection

## Abstract

**Introduction and importance:**

Renal cell carcinoma is the most lethal malignancy of urinary tract. Invasion of right lobe of liver by Renal cell carcinoma is rare and possess a treatment challenge. Simultaneous nephrectomy with right hepatectomy has been proposed as a part of multi-modality treatment approach. But its safety and feasibility is not well established.

**Case presentation:**

We herein discuss a case of 30-year old female patient who underwent simultaneous nephrectomy with right hepatectomy along with single peritoneal metastasectomy for a huge Renal cell carcinoma of right kidney and infiltrating the right lobe of liver. Intra-operatively a single peritoneal nodule was present which came positive for malignancy on frozen section. Considering young age, good performance status and oligometastatic disease definitive procedure in the form of combined right nephrectomy and right hepatectomy was performed. She was discharged from the hospital on 6th post-operative day with an uneventful post-operative course.

**Clinical discussion:**

The patients with locally advanced Renal cell carcinoma with involvement of adjacent organs require en block surgical resection in combination with targeted therapy and immunotherapy. The surgical management of patients with direct liver infiltration requires a right nephrectomy with some form of liver resection based on the extent of liver involvement to achieve a margin negative resection. In our case a plan of formal right hepatectomy was made as the tumor was infiltrating into segment VI, VII, and VIII.

**Conclusion:**

The combined nephrectomy and right hepatectomy is safe and feasible for this type of huge RCC invading right hepatic lobe.

## Highlights

• Direct infiltration of liver from a renal cell carcinoma is a rare occurrence
• Simultaneous nephrectomy with minor or major hepatectomy is required for complete resection
• It is an uncommonly performed operation and may increase the post operative morbidity
• In well selected patients this type of supra major surgery is safe and feasible if performed at tertiary care center

## 1 Introduction

Renal cell cancer (RCC) accounts for 2.2 % of all cancer diagnosis [1]. In 10 % of diagnosed cases, RCC tends to invade adjacent structures such as psoas, colon, mesentery, diaphragm, pancreas, spleen, and duodenum by the local spread [2]. For RCC involving adjacent organs, en bloc removal of kidney and involved organ is required for cancer control. Although liver is one of the most common metastatic sites, the contiguous involvement of the liver by RCC is rare and poses a diagnostic and management challenge. In cases of liver involvement, partial or sometimes a major hepatectomy provides a better chance of survival; as complete resection with clear surgical margin is necessary to achieve favourable outcome.

Simultaneous nephrectomy and major hepatectomy is an uncommonly performed surgical procedure. Nephrectomy or major hepatectomy alone are associated with substantial morbidity. The effects of combining these procedures are poorly understood because the existing knowledge comes from case reports [[3], [4], [5], [6], [7], [8]]. The most commonly cited indication in the literature is locally advanced renal cell carcinoma (RCC) with direct extension into adjacent hepatic parenchyma. Other indications for this procedure in previously reported cases are adrenocortical carcinoma, a germ cell tumor, benign cysts, renal cell carcinoma (RCC) with liver metastasis [9]. Conti et al. reported that median survival among patients having received cytoreductive nephrectomy improved from 13 to 19 months in the era of targeted therapy, while survival among patients not receiving cytoreductive nephrectomy increased slightly (from 3 to 4 months) [10].

We herein report a rare case of large RCC with direct infiltration into liver and a single peritoneal metastasis which was successfully managed by performing a right nephrectomy with right hepatectomy along with metastasectomy of peritoneal nodule. This case report is in line with the SCARE criteria [11].

## 2 Case presentation

A 30 year old female presented with complaints of pain in right upper abdomen and awareness of lump in right flank. History of loss of weight and appetite was present. There was no history of hematuria. On per abdominal examination a large lump of approx. size 15 × 12 cm was present in right upper quadrant and reaching up to right iliac fossa.

**Fig. 1:**
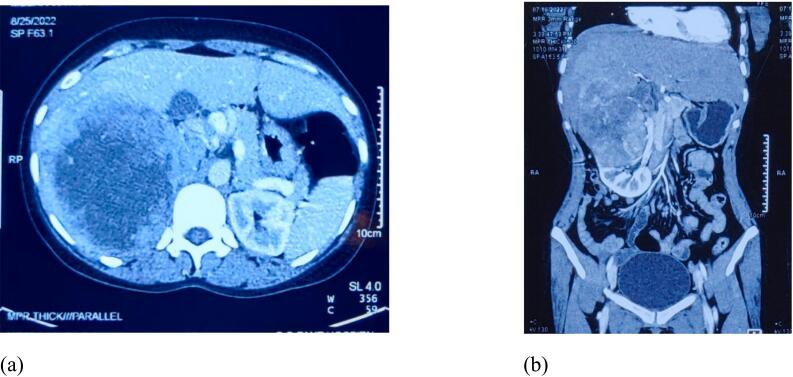
(a) A transverse and (b) a coronal CT images showed a large renal cell carcinoma, infiltrating the right lobe of liver.

Contrast enhanced computed tomography (CT) showed a large heterogeneously enhancing mass lesion of size 16.5 cm × 10.5 cm × 10 cm in hepato-renal space [Fig. 1(a) and (b)]. The lesion showed large area of central necrosis with thick nodular peripheral enhancement. Superiorly the lesion was seen to infiltrate most of the contiguous right lobe of liver (segment VI, VII and VIII). Inferiorly it was seen to involve upper pole of right kidney with sharp distinct margins. Magnetic resonance imaging (MRI) abdomen showed findings similar to CT scan with a large enhancing mass lesion in right lobe of liver with extension into right retro peritoneum and infiltrating into right kidney.

The case was discussed in multi-disciplinary tumor board meeting. A pre-operative diagnosis of locally advanced RCC with liver infiltration was made and surgical intervention with en bloc right nephrectomy and hepatectomy was planned. A reversed L incision was given from the skin to peritoneum and abdominal cavity was exposed. A 3 × 2cm size peritoneal nodule was noted near mesoappendix, it was dissected and sent for frozen section. Intra-op frozen report of the peritoneal nodule was positive for malignancy. There was no evidence of any other peritoneal or omental nodule. Considering the young age, good performance status of the patient and a single isolated peritoneal metastasis, decision of cyto-reductive surgery in the form of simultaneous nephrectomy with right hepatectomy was taken. The posterior peritoneum was opened and the lower pole of right kidney was mobilized. Right renal artery was dissected and ligated. As the mobilisation of right lobe of liver was restricted, parenchymal transection was done first. The liver parenchyma was transected using a Cavitron Ultrasonic Surgical Aspirator (CUSA) and harmonic. CVP was kept low (approx. 4 mmHg). Vascular inflow occlusion (Pringle Maneuver) was not used. The right hepatic vein was clamped, divided and ligated with prolene suture. Retroperitoneal dissection of the upper kidney was done. Right renal vein and artery were divided with an Echelon white stapler. Right ureter was ligated and divided and the combined right nephrectomy and hepatectomy was completed.

**Fig. 2:**
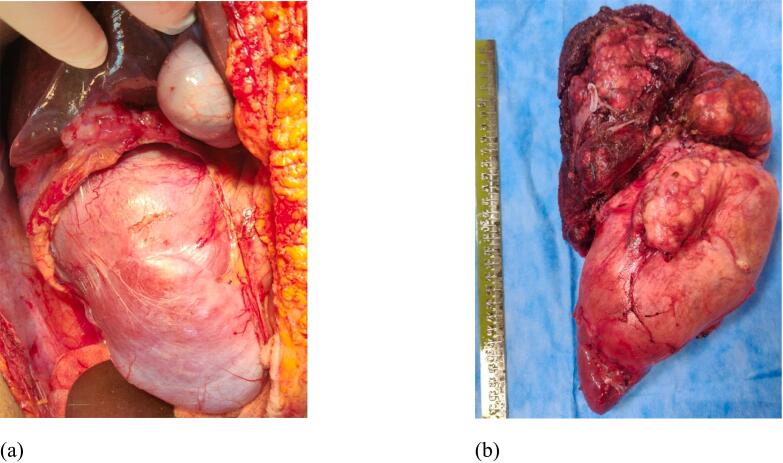
(a) and (b) macroscopic appearance of tumor arising from upper pole of right kidney and infiltrating into right lobe of liver.

Macroscopic examination revealed a 25 cm × 15 cm × 10 cm size soft to firm mass lesion, arising from the upper pole of right kidney [Fig. 2(a) & (b)]. Areas of cystic degeneration and necrosis were present. There was no breach in the renal capsule however the tumor was extending superiorly and was infiltrating the right liver (segment VI, VII, VII). Fat planes with IVC, right colon and duodenum were maintained. Multiple enlarged lymph nodes were present in para-caval region, largest measuring 2 cm × 2cm. Patient received intensive care monitoring for two days and recovered well. She was started orally on post-operative day (POD) 2 and drain was removed on POD 5. She was discharged on POD 6. The patient recovered without any hepatic or urinary complications. Post recovery she received chemotherapy and is free of local recurrence or other systemic metastasis at 12 months of follow up.

**Fig. 3:**
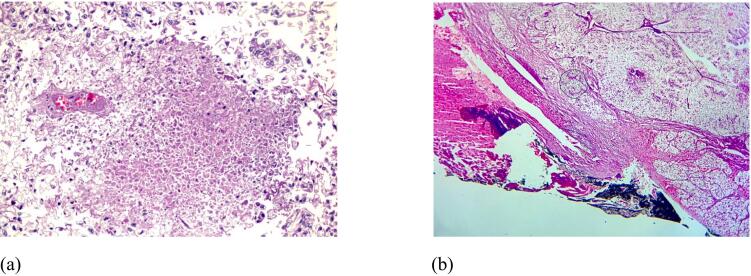
Histologic examination revealing tumor with central necrosis (a) and liver infiltration (b).

Microscopic examination revealed grade IV clear cell RCC, originating from upper pole of right kidney. Tumor was extending into renal sinus, right adrenal gland and liver. Lypmhovascular invasion was present [Fig. 3(a) & (b)]. Eight lymph nodes were dissected, all of them were involved with tumor (Final stage pT4N1M1).

## 3 Discussion

Renal cell carcinoma (RCC) is the most common type of kidney cancer in adults. It accounts for about 3 % of all cancers with a worldwide annual increase of 1.5–5.5 %. This is mainly due to enhanced detection of tumors by increased use of imaging techniques [12]. It has a highly variable natural history and biological behaviour. The survival is dependent on the stage at diagnosis. It may present as localized disease, regional or locally advanced disease and metastatic disease. Approximately 30–40 % patients with RCC will either present with or later develop metastatic disease [13].

Clear cell RCC is the most common histological subtype of RCC and accounts for 75 % of diagnoses. Other histological subtypes include papillary, chromophobe and medullary type. These subtypes have significant prognostic and treatment predictive value. Chromophobe RCC carries best prognosis but represents only 5 % of cases. Papillary RCC has better survival than clear cell as clear cell RCC are more likely to metastasize to the lungs, liver and bones through hematogenous spread.

The management approach to RCC is also guided by the stage at presentation with surgical resection being the most curative treatment for localized disease while targeted therapy and immunotherapy being the mainstay of treatment for metastatic disease. The patients with locally advanced RCC with involvement of adjacent organs require en block surgical resection in combination with targeted therapy and immunotherapy. Despite complete surgical resection they have a significant risk of local recurrence and disease progression. Unfortunately even after margin negative surgical resection the 5 year survival remains around 5 % in these patients [14]. Involvement of the liver occurs either by contiguous extension or hematogenus spread. A large renal tumor may indent or compress the liver but actual invasion is rare. Hematogenous spread is more common [15]. When intrahepatic metastases are present, 98 % of patients have other metastases as well. Most of these patients are symptomatic, however, the liver function in most are normal. Partial hepatectomy in direct extension gives a good survival.

Direct liver infiltration by RCC is both a diagnostic and management challenge. A contrast enhanced CT scan is the imaging modality of choice for the diagnosis of RCC, however in case of direct liver infiltration establishing the primary site of tumor becomes challenging. Additional imaging modalities such MRI abdomen or FDG-PET CT may be required to further confirm the diagnosis.

The surgical management of patients with direct liver infiltration by RCC requires a right nephrectomy with some form of liver resection based on the extent of liver involvement to achieve a margin negative resection. In our case a plan of formal right hepatectomy was made as the tumor was infiltrating into segment VI, VII, and VIII. Intra-operatively we also found a metastatic deposit on the parietal peritoneum but considering the good performance status of the patient and oligometastatic disease, decision to continue with surgical resection was contemplated.

In a study by Joyce et al. which included 103 patients, outcomes with no hepatic resection (n = 68) vs. with hepatic resection (n = 34) was compared. Out of 34 cases who underwent simultaneous nephrectomy with hepatic resection 50 % (n = 17) cases had a direct extension to the liver. The median follow-up was 7 years (0.5–26.7 years), with 2-year CSS and OS of 40 % vs. 29 % (HR: 0.72, P = 0.2) and 40 % vs. 28 % (HR: 0.80, P = 0.30) in hepatic resection vs no hepatic resection cohort respectively [16].

In patients with metastatic RCC there are studies supporting the role of cytoreductive surgery in association with systemic therapy compared with interferon treatment alone to achieve a significant survival benefit (13.6 months versus 7.8 months, respectively) [17,18]. Debulking of >90 % of renal cancer metastases has been associated with significantly increased survival in at least one multivariate model [19]. In this era of new systemic options (sunitinib, sorafenib, bevacizumab, and temsirolimus), a more aggressive surgical approach may be appropriate for patients with advanced RCC.

## 4 Conclusion

A locally advanced RCC may rarely present with contiguous liver infiltration. Simultaneous nephrectomy with hepatectomy with clear surgical margin provides a better chance of survival in these patients. The multimodal approach consisting of cytoreductive nephrectomy, systemic therapy (which includes cytokines or targeted molecules), and metastasectomy have been shown to be useful in prolonging the survival and improving the quality of life in a select group of patients with metastatic renal cancer. Patients with oligometastatic disease, good performance status, and delayed presentation of the secondaries have better results following this integrated approach. A combination of nephrectomy and right hepatectomy along with metastasectomy appears to be safe and feasible for well selected patients with huge renal cell carcinoma directly invading the right lobe of the liver.

## Ethical approval

Ethical approval for this case report was taken from Institutional Ethics Committee (159/IEC/MAMC/2023) Maulana Azad Medical College, New Delhi on 26 August 2023.

## Funding

None.

## Author contribution

Author 1 & 2: study concept or design, data collection, data analysis or interpretation, writing the paper.

Author 3 & 4: Revising the report and final approval.

## Guarantor

Devendra Choudhary (MS)- Corresponding author

choudhary.devendra61@gmail.com

## Consent

Written informed consent was obtained from the patient for publication of this case report and accompanying images. A copy of the written consent is available for review by the Editor-in-Chief of this journal on request.

## Conflict of interest statement

None.

## References

[1] Padala S., Barsouk A., Thandra K.A. (2020). Epidemiology of renal cell carcinoma. World J. Oncol..

[2] Manjunath S., Ramachandra C., Murthy V. (2007). Surgical resection for locally invasive renal cell carcinoma: is it worthwhile?. Indian J. Urol..

[3] Talarico F., Buli P., Iusco D. (2007). Synchronous nephrectomy and right hepatectomy for metastatic chromophobe renal cell carcinoma: report of a case and review of the literature. Chir. Ital..

[4] Wong J.A., Whelan T., Morse M. (2006). Radical nephrectomy with en bloc resection of liver, diaphragm, and lung for locally invasive sarcomatoid renal cell carcinoma. Urology.

[5] Johnin K., Nakai O., Kataoka A. (2001). Surgical management of renal cell carcinoma invading into the liver: radical nephrectomy en bloc with right hepatic lateral sector. Urology.

[6] Fujisaki S., Takayama T., Shimada K. (1997). Hepatectomy for metastatic renal cell carcinoma. Hepatogastroenterology.

[7] Okajima E., Ozono S., Nagayoshi J. (1994). A case report of synchronous triple cancer resected simultaneously. Jpn. J. Clin. Oncol..

[8] Dineen M.K., Pastore R.D., Emrich L.J., Huben R.P. (1988). Results of surgical treatment of renal cell carcinoma with solitary metastasis. J. Urol..

[9] Yezhelyev M., Master V., Egnatashvili V., Kooby D.A. (2009). Combined nephrectomy and major hepatectomy: indications, outcomes, and recommendations. J. Am. Coll. Surg..

[10] Conti S.L., Thomas I.C., Hagedorn J.C. (2014). Utilization of cytoreductive nephrectomy and patient survival in the targeted therapy era. Int. J. Cancer.

[11] Agha R.A., Franchi T., Sohrab C., Mathew G., Kirwan A., Thomas A. (2020). The SCARE 2020 guideline: updating consensus Surgical Case Report (SCARE) guidelines. Int. J. Surg..

[12] Chow W.H., Devesa S.S., Warren J.L. (1999). The rising incidence of renal cell carcinoma in the United States of America. JAMA.

[13] Shvarts O., Lepper J.T., Figlin R.A., Belldegrun A.S. (2005). Renal cell carcinoma 2005, new frontiers in staging, prognostication and targeted therapy. J. Urol..

[14] Wein J., Kavoussi L., Novick A., Partin A. (2012).

[15] DeKernion J.B., Walsh P.C., Gittes R.F., Perlmutter A.D., Stamey T.A. (1986). W: Campbells Urology, t. 2, red.

[16] Joyce D., Psutka S., Groeschl R. (2017). Complications and outcomes associated with surgical management of renal cell carcinoma involving the liver: a matched cohort study. Urology.

[17] Flanigan R.C., Salmon S.E., Blumenstein B.A. (2001). Nephrectomy followed by interferon alfa-2b compared with interferon alfa-2b alone for metastatic renal-cell cancer [see comment]. N. Engl. J. Med..

[18] Flanigan R.C., Mickisch G., Sylvester R. (2004). Cytoreductive nephrectomy in patients with metastatic renal cancer: a combined analysis. J. Urol..

[19] Pierorazio P.M., McKiernan J.M., McCann T.R. (2007). Outcome after cytoreductive nephrectomy for metastatic renal cell carcinoma is predicted by fractional percentage of tumour volume removed. BJU Int..

